# Left ventricular global function index assessed by cardiovascular magnetic resonance for the prediction of cardiovascular events in ST-elevation myocardial infarction

**DOI:** 10.1186/s12968-015-0161-x

**Published:** 2015-07-16

**Authors:** Ingo Eitel, J. Pöss, A. Jobs, C. Eitel, S. de Waha, J. Barkhausen, S. Desch, H. Thiele

**Affiliations:** University Heart Center Lübeck, Medical Clinic II (Cardiology/Angiology/Intensive Care Medicine), University Hospital Schleswig-Holstein, Ratzeburger Allee 160, 23538 Lübeck, Germany; Department of Radiology, University of Luebeck, Luebeck, Germany

**Keywords:** Cardiovascular magnetic resonance, Myocardial infarction, Prognosis, Left ventricular ejection fraction, Left ventricular global function index

## Abstract

**Background:**

The left ventricular performance index (LVGFI) as a comprehensive marker of cardiac performance integrates LV structure with global function within one index. In a prospective cohort study of healthy individuals the LVGFI demonstrated a superior prognostic value as compared to LV ejection fraction (LVEF). In patients after ST-segment elevation myocardial infarction (STEMI), however, the role of the LVGFI is unknown. Aim of this study was to investigate the relationship between the LVGFI and infarct characteristics as well as prognosis in a large multicenter STEMI population.

**Methods:**

In total 795 STEMI patients reperfused by primary angioplasty (<12 h after symptom onset) underwent cardiovascular magnetic resonance (CMR) at 8 centers. CMR was completed within one week after infarction using a standardized protocol including LV dimensions, mass and function for calculation of the LVGFI. The primary clinical endpoint of the study was the occurrence of major adverse cardiac events (MACE).

**Results:**

The median LVGFI was 31.2 % (interquartile range 25.7 to 36.6). Patients with LVGFI < median had significantly larger infarcts, less myocardial salvage, a larger extent of microvascular obstruction, higher incidence of intramyocardial hemorrhage and more pronounced LV dysfunction (*p* < 0.001 for all). MACE and mortality rates were significantly higher in the LVGFI < median group (*p* < 0.001 and *p* = 0.003, respectively). The LVGFI had an incremental prognostic value in addition to LVEF for prediction of all-cause mortality.

**Conclusions:**

The LVGFI strongly correlates with markers of severe myocardial and microvascular damage in patients with STEMI, offering prognostic information beyond traditional cardiac risk factors including the LVEF.

**Trials registration:**

ClinicalTrials.gov: NCT00712101

## Background

Myocardial damage following an acute myocardial infarction (MI) is accompanied by significant changes in left ventricular (LV) mechanical function and myocardial structure [1]. Numerous studies have demonstrated that the measurement of LV ejection fraction (LVEF) as a marker of global systolic myocardial function is a powerful predictor of morbidity and mortality in patients with acute reperfused MI [2, 3]. Consequently, the LVEF has become an important prognostic and functional marker for routine risk stratification and therapeutic decision making [4]. However, the use of the LVEF has several limitations including the lack of information on LV size or mass as well as on diastolic function. Importantly, LV mass and other structural LV parameters have been also shown to be prognostic relevant in various cardiovascular diseases including MI [5, 6].

Recently, a novel LV global function index (LVGFI) that integrates LV structure with global function has been introduced as a marker for comprehensive characterization of cardiac performance. In a multicenter prospective cohort study of healthy individuals, the LVGFI was independently associated with the occurrence of hard cardiovascular events and had a superior prognostic value as compared to LVEF [7]. In patients after acute MI only one small single-center study assessed the relation between the LVGFI and infarct characteristics, however, the prognostic role of the LVGFI after reperfused AMI is completely unknown [8].

Cardiovascular magnetic resonance (CMR) has emerged as the gold standard for measuring LVEF, LV volumes and mass. Moreover, other clinically important infarct characteristics including infarct size (IS) and reperfusion injury (microvascular obstruction [MO]) can be visualized, providing a comprehensive assessment of patients sustaining myocardial injury [9]. Therefore, CMR is ideally suited for the assessment of the LVGFI and its relation with myocardial damage.

The aim of our study was to investigate the relationship between the LVGFI and infarct characteristics as well as prognosis in a large multicenter ST-segment elevation MI (STEMI) population treated with primary percutaneous coronary intervention (PCI).

## Methods

### Study design

This prospective CMR study was a predefined substudy of the AIDA STEMI (Abciximab Intracoronary versus intravenously Drug Application in STEMI) trial, which compared intravenous versus intracoronary abciximab application in STEMI patients and did not show a difference in IS, reperfusion injury and clinical outcome between the treatment groups. The detailed design and main results of the trial have previously been published [10, 11]. Briefly, AIDA STEMI was a randomized, open-label, multicenter trial. Patients presenting with STEMI in the first 12 h after symptom onset were randomly assigned in a 1:1 ratio by a central web-based randomization system to intracoronary versus intravenous abciximab bolus (0.25 mg/kg bodyweight) during primary PCI with a subsequent 12 h intravenous infusion at 0.125 μg/kg/min (maximum 10 μg/min).

Patients were enrolled at 22 sites in Germany, with a final enrolled trial population of 2.065 patients (intracoronary abciximab [*n* = 1.032], intravenous abciximab [*n* = 1.033]). The study was approved by national regulatory authorities and Ethical committee of the University of Leipzig. All patients provided written informed consent. This trial is registered with ClinicalTrials.gov, NCT00712101.

Consecutive patients enrolled in the AIDA STEMI trial at 8 sites were included in the CMR substudy [12]. The sites were chosen based on proven expertise in performing CMR examinations in patients with MI. By protocol CMR was performed on days 1 to 10 after the index event for assessment of myocardial salvage, IS, presence and extent of MO, LVEF, and endsystolic and enddiastolic volumes. Exclusion criteria for the CMR substudy were the typical contraindications for CMR as described previously [10, 11].

The detailed scan protocol on a clinical 1.5 or 3.0 Tesla MR scanner has been described previously [10–12]. CMR images were sent on storable media to the CMR core laboratory for assessment by fully blinded operators.

### Image analysis

For all quantitative analyses, certified CMR evaluation software was used (cmr42 Circle Cardiovascular Imaging Inc, Calgary, Alberta, Canada). LV enddiastolic (LVEDV) and endsystolic volumes (LVESV) as well as LV mass were assessed according to standard definitions from functional images. Semiautomated computer-aided threshold detection was used to identify regions of edema, MO and infarcted myocardium. A myocardial region was regarded as affected if at least 10 adjacent myocardial pixels revealed a signal intensity of >2 standard deviations (SD) of remote myocardium for edema and >5 SD in late gadolinium enhancement (LGE) images [12]. The hypointense signal within the area of increased T2-signal intensity which represents hemorrhage was included in area at risk assessment. IS, area at risk, and MO were expressed as percentage of LV volume (%LV). MO – if present – was included into the overall IS analysis and was additionally quantified separately. Salvaged myocardium was quantified as the difference between the volume of increased T2-signal (area at risk) and the volume of LGE (IS), as previously described [13]. The CMR core laboratory is highly experienced in CMR acquisition and post-processing with excellent reproducibility and low inter- and intraobserver variability for IS and myocardial salvage assessment [14, 15].

### Calculation of the LVGFI

The LV stroke volume (LVSV) was calculated by LVEDV-LVESV. The LV global volume was defined as the sum of the LV mean cavity volume ([(LVEDV + LVESV)/2] and the myocardium volume. LV myocardial volume was calculated as LV myocardial mass divided through the specific myocardial density (1.05 g/mL). The LVGFI was thus defined according to the following established formula and expressed as a percentage: [7] LVGFI = (LVSV/LV global volume)*100.

### Clinical endpoints

The primary clinical endpoint of this substudy was a composite of all-cause death, reinfarction, and new congestive heart failure within one year after infarction. All components of the combined clinical endpoint were adjudicated by a clinical endpoints committee, blinded to the patient's assigned treatment, based on data provided by the clinical trial sites. To avoid double counting of patients with more than one event, each patient contributed only once to the composite major adverse cardiovascular event (MACE) endpoint (death > reinfarction > congestive heart failure). Detailed outcome definitions have been reported previously [10–12].

### Statistical analysis

Patients were grouped by the median of the LVGFI into a less than the median and a median or above LVGFI group. Baseline patient characteristics, procedural details, and CMR findings are described according to the median of the LVGFI. Data for continuous variables are presented in medians with 25th and 75th percentiles. Categorical variables are presented as frequencies and percentages. Differences between groups were assessed by Fisher’s exact or the chi-square test for categorical variables and by the Student’s *t* test for continuous data with normal distribution. Otherwise the non-parametric Wilcoxon rank-sum test was used. Hazard ratios (HR) with 95 % confidence intervals were calculated for binary outcomes. Univariate and stepwise multivariate Cox regression analyses were performed to identify predictors of MACE. Multivariate regression was performed using only variables with a p-value <0.05 in univariate regression analyses. For univariate analyses, all variables of Table 1 were considered. To ensure statistical robustness of the Cox regression model we included the TIMI-risk score instead of the individual risk components (age, diabetes, hypertension, heart rate, Killip-class, weight, anterior infarction, and time to treatment) to reduce the number of analyzable parameters with respect to our sample size and total number of clinical events. To make the HR comparable, the continuous variables LVGFI and LVEF were dichotomized according to the median. Myocardial salvage, IS and MO were not included in the Cox regression model as the main purpose of this study was to identify an independent predictive value of the LVGFI in comparison to the LVEF.

**Table 1:** Patient characteristics

Variable	Total study	LVGFI ≥ median	LVGFI < median	*p*-value
	*N* = 795	*n* = 397	*n* = 398	
Age (years)	62 (51 – 71)	61 (51 – 70)	63 (51 – 71)	0.40
Male sex: n (%)	603 / 795 (76 %)	283 / 397 (71 %)	320 / 398 (80 %)	0.003
Cardiovascular risk factors: n (%)				
Current smoking	339 / 727 (47 %)	169 / 359 (47 %)	170 / 368 (46 %)	0.81
Hypertension	540 / 792 (68 %)	256 / 394 (65 %)	284 / 398 (71 %)	0.05
Hypercholesterolemia	304 / 787 (39 %)	152 / 391 (39 %)	152 / 396 (38 %)	0.88
Diabetes mellitus		77 / 395 (20 %)	83 / 397 (21 %)	0.62
BMI (kg/m^2^)	27.3 (24.9 – 30.3)	27.7 (24.9 – 30.4)	27.7 (24.8 – 30.3)	0.48
Previous infarction: n (%)	48 / 794 (6 %)	18 / 396 (5 %)	30 / 398 (8 %)	0.08
Anterior infarction: n (%)	363 / 758 (48 %)	127 / 377 (34 %)	236 / 381 (62 %)	<0.001
Times (min)				
Symptom-onset to PCI hospital admission	180 (109 – 310)	160 (100 – 285)	190 (116 – 346)	0.005
Door-to-balloon-time	30 (22 – 42)	30 (22 – 42)	29 (22 – 42)	0.28
Killip-class on admission: n (%)				<0.001
1	699 / 795 (88 %)	373 / 397 (94 %)	326 / 398 (82 %)	
2	59 / 795 (7 %)	15 / 397 (4 %)	44 / 398 (11 %)	
3	20 / 795 (3 %)	4 / 397 (1 %)	16 / 398 (4 %)	
4	17 / 795 (2 %)	5 / 397 (1 %)	12 / 398 (3 %)	
Number of diseased vessels: n (%)				0.18
1	422 / 795 (53 %)	225 / 397 (57 %)	197 / 398 (50 %)	
2	225 / 795 (28 %)	107 / 397 (27 %)	118 / 398 (30 %)	
3	148 / 795 (19 %)	65 / 397 (16 %)	83 / 398 (21 %)	
Infarct related artery: n (%)				<0.001
Left anterior descending	347 / 795 (44 %)	117 / 397 (30 %)	230 / 398 (58 %)	
Left circumflex	97 / 795 (12 %)	51 / 397 (13 %)	46 / 398 (12 %)	
Right coronary artery	344 / 795 (12 %)	227 / 397 (57 %)	117 / 398 (29 %)	
Left main	5 / 795 (1 %)	2 / 397 (1 %)	3 / 398 (1 %)	
Bypass graft	2 / 795 (<1 %)	0 / 397 (0 %)	2 / 398 (1 %)	
TIMI-flow before PCI: n (%)				0.001
TIMI-flow 0	445 / 795 (56 %)	204 / 397 (51 %)	241 / 398 (61 %)	
TIMI-flow I	104 / 795 (13 %)	46 / 397 (12 %)	58 / 398 (15 %)	
TIMI-flow II	129 / 795 (16 %)	70 / 397 (18 %)	59 / 398 (15 %)	
TIMI-flow III	117 / 795 (15 %)	77 / 397 (19 %)	40 / 398 (10 %)	
Stent implanted: n (%)	777 / 795 (98 %)	388 / 397 (98 %)	389 / 398 (98 %)	0.94
Thrombectomy: n (%)	190 / 795 (24 %)	88 / 397 (22 %)	102 / 398 (26 %)	0.25
TIMI-flow post PCI: n (%)				0.12
TIMI-flow 0	12 / 794 (2 %)	4 / 397 (1 %)	8 / 397 (2 %)	
TIMI-flow I	104 / 795 (13 %)	5 / 397 (1 %)	14 / 397 (4 %)	
TIMI-flow II	62 / 794 (8 %)	31 / 397 (8 %)	31 / 397 (8 %)	
TIMI-flow III	701 / 794 (88 %)	357 / 397 (90 %)	344 / 397 (87 %)	
TIMI-risk score	3 (2 – 5)	3 (2 – 4)	4 (2 – 5)	<0.001
ST-segment resolution	54 (21 – 78)	63 (28 – 81)	49 (14 – 72)	0.02
Intraaortic balloon pump: n (%)	35 / 795 (4 %)	10 / 397 (3 %)	25 / 398 (6 %)	0.01
Concomitant medications: n (%)				
ß-blockers	759 / 793 (96 %)	376 / 396 (95 %)	383 / 397 (97 %)	0.29
ACE-inhibitors/AT-1-antagonist	754 / 793 (95 %)	378 / 396 (96 %)	376 / 397 (95 %)	0.63
Aspirin	795 / 795 (100 %)	397 / 397 (100 %)	398 / 398 (100 %)	
Clopidogrel, prasugrel or both	775 / 77 % (100 %)	397 / 397 (100 %)	398 / 398 (100 %)	
Statins	752 / 793 (95 %)	382 / 396 (97 %)	370 / 397 (93 %)	0.04
Aldosterone antagonist	49 / 393 (13 %)	15 / 396 (4 %)	76 / 397 (19 %)	<0.001
Abciximab	748 / 794 (94 %)	370 / 397 (93 %)	378 / 397 (95 %)	0.91

Continuous data are presented as median and interquartile range

LVGFI = left ventricular global function index, ACE = angiotensin-converting enzyme, AT-1 = angiotensin1, BMI = body mass index, CABG = coronary artery bypass graft, CMR = cardiovascular magnetic resonance, IQR = interquartile range, Med = Median, PCI = primary percutaneous coronary intervention, TIMI = Thrombolysis in Myocardial Infarction

The incremental additive information of the LVGFI over LVEF was assessed using c-statistics. C-statistic results were compared using the nonparametric method previously described by De Long et al. [16]. A two-tailed p-value of <0.05 was defined as statistically significant. SPSS version 21.0 was used for statistical analyses.

## Results

**Fig. 1:**
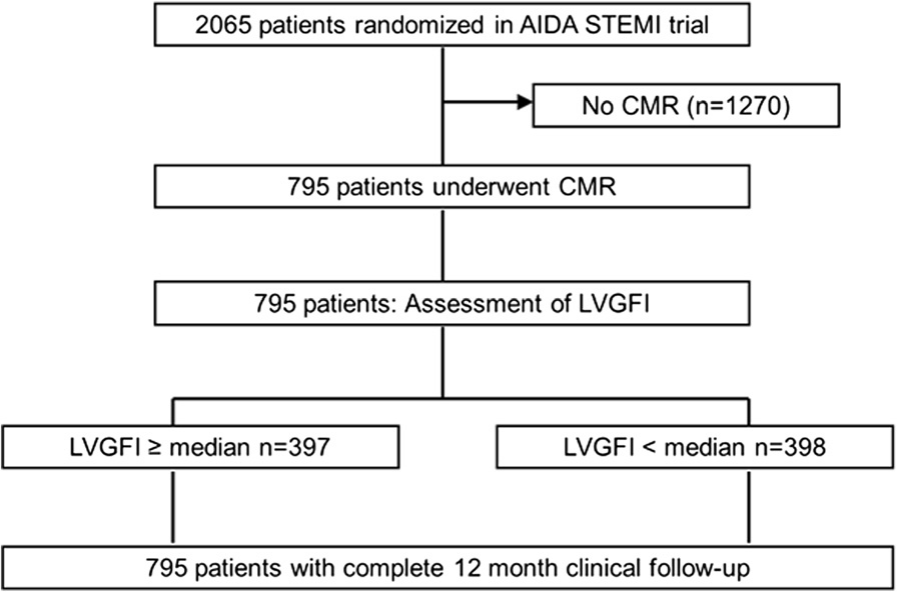
Study flow

From July 2008 to April 2011, 795 patients were enrolled in the AIDA STEMI CMR substudy (Fig. 1). In all patients, image quality was sufficient to calculate the LVGFI. Clinical outcome data 12 month after infarction were available in all patients.

### Patient characteristics

Baseline characteristics of the total study population and their association with the LVGFI are displayed in Table 1. The median age of the overall enrolled population was 62 (interquartile range, 51–71) years, and 603 (76 %) patients were men. Patients with a LVGFI < median were significantly more often male (*p* = 0.003), and were more likely to have hypertension (*p* = 0.05), anterior infarctions (*p* < 0.001), or to present with a higher Killip-class (*p* < 0.001). Consequently, the TIMI-risk score was significantly higher in the LVGFI < median group (*p* < 0.02).

### Procedural data, angiographic analysis and ST-segment resolution

Symptom-onset to reperfusion time was significantly longer in patients with LVGFI < median. Use of thrombectomy devices or stents were similar between groups. Patients with LVGFI < median had significantly more often left anterior descending culprit lesions (*p* < 0.001). There was also a significantly impaired TIMI-flow grade before PCI (*p* = 0.001) and ST-segment resolution (*p* = 0.02) after PCI in patients with LVGFI < median.

### CMR findings

**Fig. 2:**
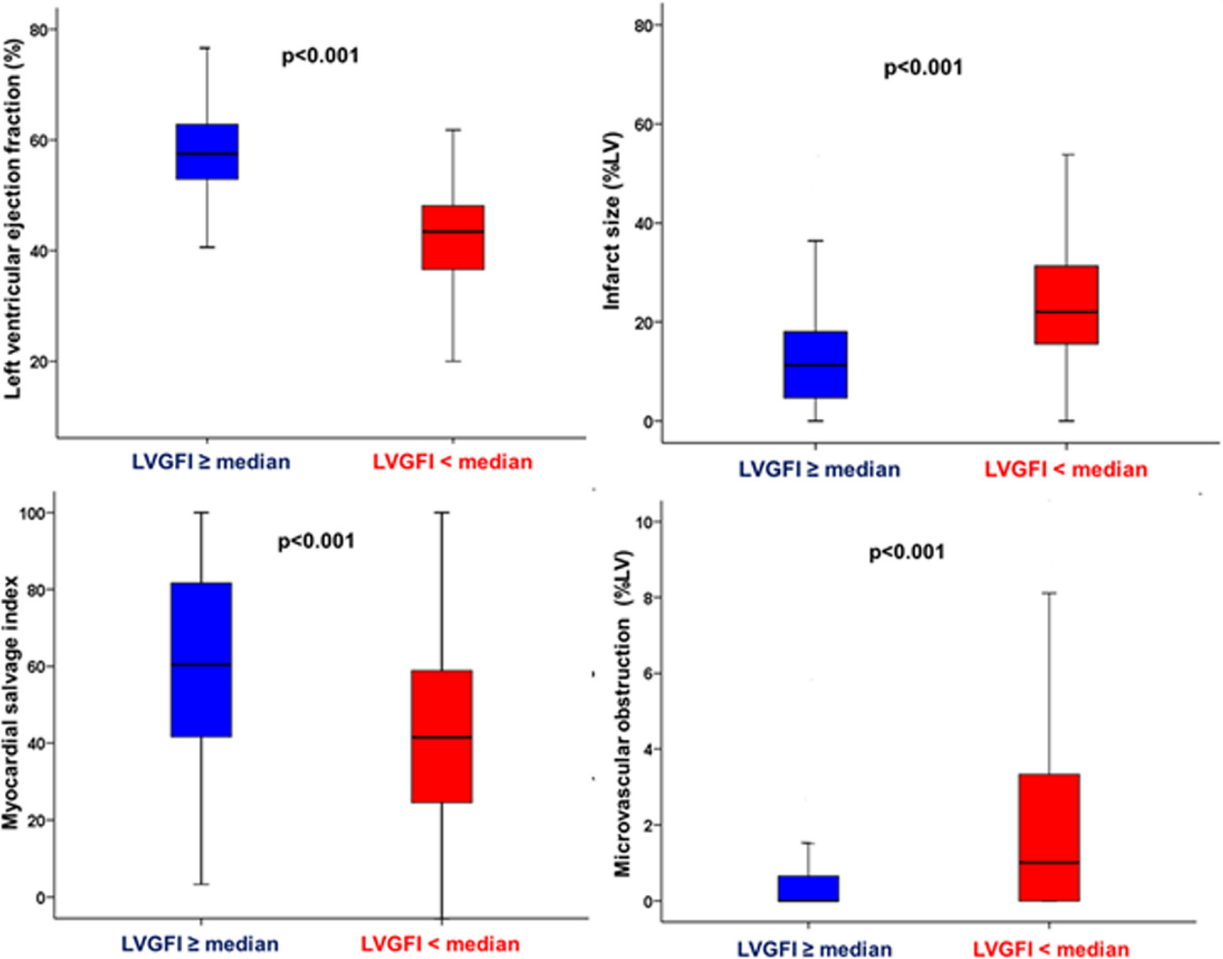
Box (25^th^ percentile, median and 75^th^ percentile) and whisker (10^th^ and 90^th^ percentiles) plots of LVEF, infarct size, myocardial salvage index and microvascular obstruction according to the LVGFI

The median time between the index event and CMR was 3 days (interquartile range, 2–4 days) for both LVGFI groups (*p* = 0.70). In all patients, the median LVGFI was 31.2 % (interquartile range 25.7 to 36.6). Patients with LVGFI < median had significantly larger infarcts, less myocardial salvage, a larger extent of MO, higher incidence of intramyocardial hemorrhage and more pronounced LV dysfunction (Fig. 2, Table 2).

**Table 2:** Cardiovascular magnetic resonance results

Characteristic	Total study	LVGFI ≥ median	LVGFI < median	*p*
	*N* = 795	*n* = 397	*n* = 398	
Area at risk (Edema) (%LV)	35 (25 – 48)	30 (21 – 41)	41 (30 – 53)	<0.001
Infarct size (%LV)	17 (8 – 25)	11 (5 – 18)	22 (16 – 31)	<0.001
Myocardial salvage index	51 (33 – 69)	60 (42 – 82)	42 (24 – 59)	<0.001
Late MO (%LV)	0 (0 – 1.8)	0 (0 – 0.6)	1.1 (0 – 3.3)	<0.001
Hypointense core (%LV) (Hemorrhage)	0 (0 – 1.4)	0 (0 – 0.0)	0.5 (0 – 3.3)	<0.001
LV ejection fraction (%)	51 (43 – 58)	58 (53 – 63)	43 (37 – 48)	<0.001
LV enddiastolic volume (mL)	146 (120 – 173)	140 (114 – 165)	151 (128 – 180)	<0.001
LV endsystolic volume (mL)	72 (54 – 91)	59 (45 – 75)	86 (67 – 108)	<0.001
LV mass (%LV)	131 (109 – 156)	120 (100 – 144)	143 (122 – 167)	<0.001

Data are presented as median and interquartile range

LVGFI = left ventricular global function index; CMR = cardiovascular magnetic resonance, LV = left ventricular, MO = microvascular obstruction

The area at risk, LV volumes (LVEDV and LVESV) as well as LV mass were also significantly larger in patients with a LVGFI < median (Table 2).

**Fig. 3:**
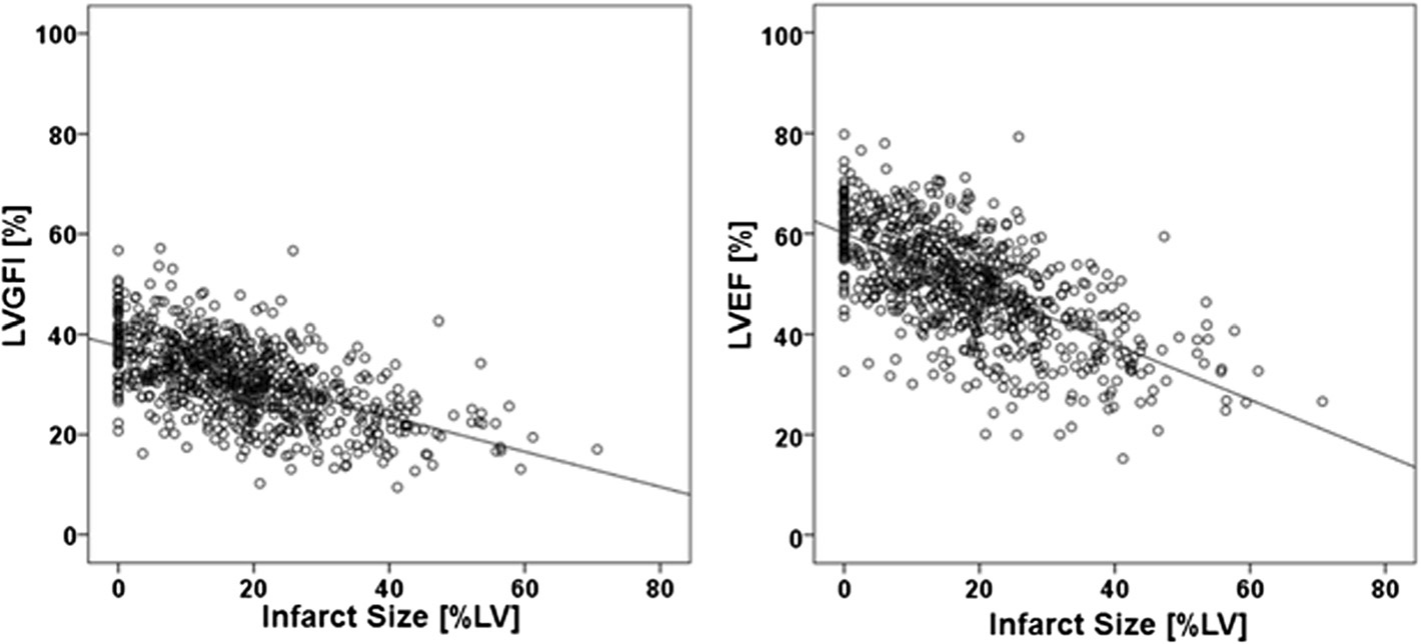
Linear correlation between infarct size and LVGFI as well as infarct size and LVEF

The extent of infarcted myocardium was inversely related to LVGFI (r = -0.56, *p* < 0.001) and LVEF (r = -0.50, *p* < 0.001) (Fig. 3).

**Fig. 4:**
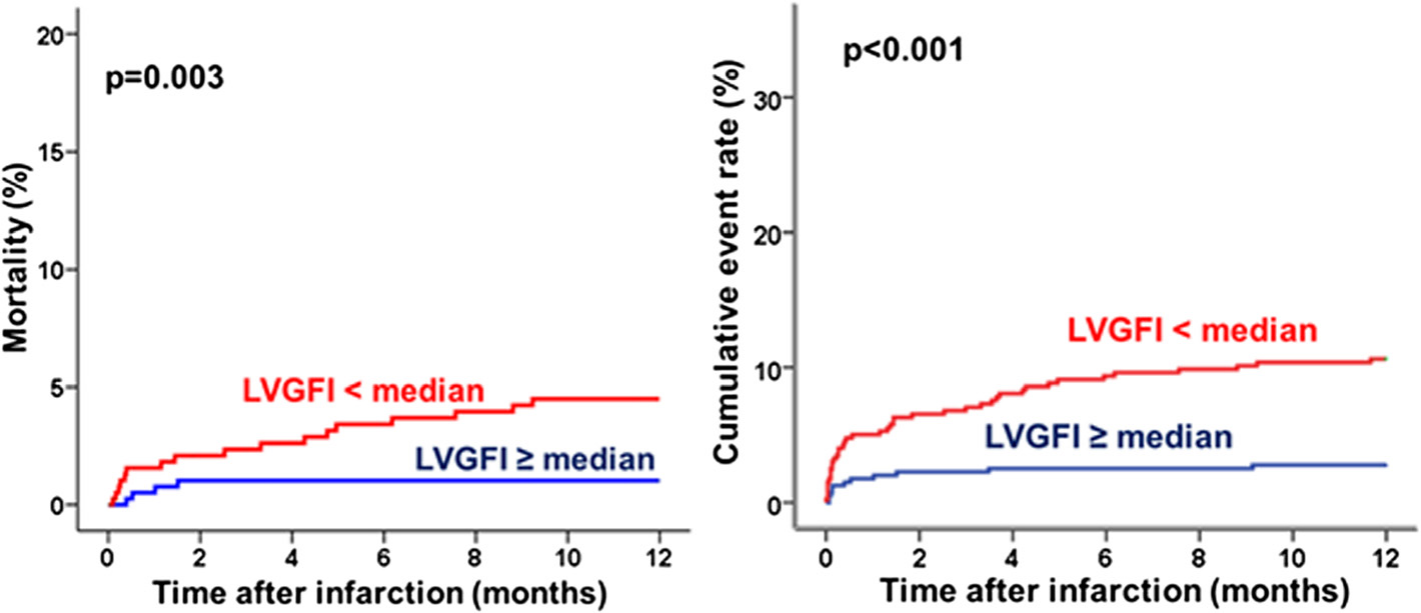
Kaplan-Meier curve of the incidence of death and major adverse cardiovascular events (death, reinfarction, readmission for heart failure) during 1-year follow-sup

### Predictors of the LVGFI

Multivariate analyses were performed to assess independent predictors of the LVGFI. In our regression model adjusted for significant variables in univariable regression analysis using the LVGFI as the dependent variable, male sex, symptom-onset to reperfusion time, culprit lesion left anterior descending, Killip-class on admission and IS were the strongest predictors of the LVGFI (Table 3).

**Table 3:** Univariable and multivariable linear regression analysis for the prediction of the LVGFI

	LVGFI			
	Univariable		Multivariable	
Variable	*ß*	P	*ß*	P
Sex	-0.11	0.02	-0.13	<0.001
Previous infarction	0.08	0.03	-	-
Anterior myocardial infarction	-0.24	<0.001	-	-
Killip-class on admission	-0.22	<0.001	-0.11	0.002
Symptom-onset-to-reperfusion (min)	-0.10	0.007	0.22	0.005
Number of diseased vessel	-0.13	<0.001	-	-
Culprit lesion = LAD	-0.25	<0.001	-0.11	0.001
TIMI-flow grade before PCI	0.19	<0.001	-	-
TIMI-flow grade after PCI	0.08	0.03	-	-
Thrombectomy	0.08	0.03	-	-
Intraaortic balloon pump	0.12	0.001	-	-
Area at risk (%LV)	-0.32	<0.001	-	-
Infarct size (%LV)	-0.54	<0.001	-0.46	<0.001

PCI = percutaneous coronary intervention; LVGFI = left ventricular global function index

### LVGFI and clinical outcome

At 12-month follow-up, there were 53 clinical events. MACE and mortality rates were significantly higher in the LVGFI < median group. Consequently, patients who died had a significantly lower LVGFI (23.4, interquartile range 16.7 to 27.9 versus 31.3, interquartile range 25.9 to 36.8, *p* < 0.001). Figure 4 depicts Kaplan–Meier plots showing the risk of cardiac events and mortality, stratified by the LVGFI.

In addition to CMR variables, several established markers of increased patient risk were associated with an increased MACE rate at one year follow-up by simple Cox regression analysis (Table 4). Using stepwise multiple Cox regression analysis, only the TIMI-risk score and LVGFI < median emerged as independent predictors of MACE (Table 4).

**Table 4:** Predictors of MACE in univariate and multivariate Cox regression analysis

	Univariate		Stepwise multivariate	
Variable	Hazard ratio (95 % CI)	*P*	Hazard ratio (95 % CI)	*P*
Smoker	2.17 (1.14-4.13)	0.02	-	-
Number of diseased vessel	1.34 (1.01-1.79)	0.04	-	-
Peak CK	1.01 (1.00-1.02)	0.03	-	-
TIMI-risk score	1.43 (1.27-1.58)	<0.001	1.35 (1.19-1.53)	<0.001
LV ejection fraction < median	3.18 (1.70-5.95)	<0.001	-	-
LVGFI < median	3.95 (2.03-7.67)	<0.001	2.84 (1.34-6.00)	0.006

CI = confidence interval, CK = creatine kinase, LV = left ventricular, LVGFI = left ventricular global function index, TIMI = Thrombolysis In Myocardial Infarction

Myocardial salvage, IS and MO were not included in the Cox regression model as the main purpose of this study was to identify an independent predictive value of the LVGFI in comparison to the LVEF

The hazard ratio for the LVGFI and LVEF as continuous variable were 0.92 (confidence interval 0.89 to 0.95) and 0.93 (confidence interval 0.91 to 0.96) respectively.

C-statistic demonstrated that inclusion of the LVGFI in addition to LVEF for prediction of mortality resulted in a significant increase of the c-statistics, thus demonstrating an additive prognostic value of the LVGFI over LVEF (Table 5). There was no significant increase with an inclusion of the LVGFI in addition to the LVEF for the other single clinical endpoints (reinfarction, heart failure) and MACE.

**Table 5:** C-Statistics: Additive prognostic value of the LVGFI over LVEF

Variable	C-statistic/AUC (95 % CI)	*P*
MACE:		
LVGFI	0.685 (0.651-0.717)	0.25
LVEF	0.707 (0.774-0.739)	
Mortality:		
LVGFI	0.732 (0.700-0.763)	0.05
LVEF	0.652 (0.617-0.685)	
Reinfarction:		
LVGFI	0.680 (0.646-0.712)	0.19
LVEF	0.654 (0.619-0.687)	
Heart failure:		
LVGFI	0.691 (0.658-0.723)	0.13
LVEF	0.734 (0.701-0.764)	

AUC = area under the curve, CI = confidence interval, LVGFI = left ventricular global function index

LVEF = left ventricular ejection fraction, MACE = major adverse cardiac events

## Discussion

This large multicenter CMR study is the first that evaluates the prognostic significance of the LVGFI in patients with acute reperfused STEMI. The main findings can be summarized as follows: First, there was a marked reduction of the mean LVGFI in STEMI patients. Second, a significant relation between the LVGFI and the incidence of future cardiovascular events was observed. Third, the LVGFI has an incremental prognostic value in addition to LVEF for prediction of all-cause mortality. Therefore, these results highlight the importance of a comprehensive assessment of cardiac performance including LV function and LV mass following MI and argue for their routine assessment in patients with MI.

### Determinants of the LVGFI

Based on limitations of the measurement of systolic function (LVEF), investigators have developed a new index which integrates global function (LVEF) with heart size, including LV mass [7]. In patients after acute MI, the LVGFI has only been investigated in one pooled retrospective, single-center, observational study [8]. The prognostic value of the LVGFI after acute reperfused STEMI has not been investigated yet. By reflecting functional and structural myocardial changes occurring after MI, this new marker of cardiac performance may be valuable in this setting [17].

In line with the data of Reinstadler et al. [8] we found a marked reduction of the mean LVGFI in STEMI patients when compared with a healthy multiethnic population (42 ± 6 % in the Multi-Ethnic Study of Atherosclerosis [MESA], versus 31 ± 8 % in the current study) [7]. This reduction is mainly explained by the strong correlation of the LVGFI with markers of severe myocardial and microvascular damage. Patients with impaired LVGFI had larger infarcts, less myocardial salvage and significantly larger areas of MO. Large infarcts are associated with reduced stroke volumes thereby impacting the LVGFI, whereas MO as a marker of severe microvascular injury impacts the LVGFI by increased LV volumes with subsequent adverse remodeling [18].

### Prognostic significance of the LVGFI

The LVGFI is appealing because it is a simple concept and integrates prognostically important parameters. Although the LVGFI is strongly related to LVEF, it carries additional information by accounting for ventricular mass and hypertrophy. Previous studies have shown that the risk of death or MACE is significantly increased in the presence of LV hypertrophy in patients with hypertension, coronary artery disease, or MI [5, 6]. Cardiac hypertrophy leads to various myocardial functional abnormalities and increased diastolic stiffness including shifts toward glycolytic metabolism, disorganization of the sarcomere, alterations in calcium handling, changes in contractility, loss of myocytes with fibrotic replacement, systolic and diastolic dysfunction, and electrical remodeling [5]. Despite the reported prognostic role of LV hypertrophy after STEMI, little attention has been paid to the interplay among LV hypertrophy, function, remodeling, and subsequent future cardiovascular events after MI. Interestingly, from our data it cannot be determined if LV hypertrophy was related to previous long-standing arterial hypertension with subsequent LV hypertrophy or by acute changes induced by myocardial edema as a consequence of the ischemic event itself. It has been well described that MI itself can induce acute changes in LV wall thickness in the related regions [19].

The current study clearly demonstrates for the first time that the LVGFI is associated with significantly increased MACE rates in patients with STEMI. Therefore, these data underscore the importance of the LVGFI as a marker of poor outcome in the post-MI phase. The LVGFI is correlated with established prognostic markers, such as delayed reperfusion, severe myocardial damage (IS), and reperfusion injury (MO), and might carry prognostic information of these outcome markers [20]. However, the LVGFI remained an independent predictor of excess MACE in our multivariable Cox regression analysis even after adjustment for these established prognostic markers. It is also noteworthy that the LVGFI had an incremental prognostic value in addition to the LVEF for prediction of mortality. Consequently, the LVGFI represents an important parameter for understanding the important role of cardiac performance including both LV structure and global function, thereby offering prognostic information beyond that provided by the evaluation of traditional cardiac risk factors including the LVEF.

### Clinical implications

Our work emphasizes the important association between myocardial function, structure and cardiovascular outcomes. Thus, aggressive medical heart failure therapy (e.g. including aldosterone antagonists) may be started in patients with decreased LVGFI but yet preserved LVEF. Moreover, our findings may help to identify novel treatment targets and potentially more powerful efficacy markers (e.g. in hypertension and postinfarction trials) to reduce the incidence of cardiovascular events.

### Limitations

Our study has several limitations worth emphasizing. First, our results are based on relatively small patient numbers, despite being the largest CMR study to date assessing the prognostic impact of the LVGFI. Second, the optimal time point for assessment of the LVGFI is unknown. We cannot exclude the possibility that later assessment of the LVGFI (e.g. 1 or 3 months after infarction) would have resulted in an even better prognostic value. Moreover, the LVGFI might change over time after reperfusion because of ongoing remodeling processes. Third, although T2-weighted CMR approaches for assessing the myocardium at risk are successfully applied in clinical settings and studies, some questions have been raised regarding physiological and mechanistic assumptions underlying this application [21, 22]. Therefore the presented data regarding the area at risk and myocardial salvage should be considered cautiously. Finally, the study includes only STEMI patients able to undergo a CMR scan. The exclusion of potentially sicker patients (e.g. with cardiogenic shock) with subsequent lacking CMR exam could possibly have influenced the study results.

## Conclusions

In conclusion, the LVGFI strongly correlates with markers of severe myocardial and microvascular damage in STEMI with superior prognostic information beyond traditional cardiac risk factors including the LVEF.
